# Depressive, Anxiety, and Stress Symptoms Among Schoolgirls With Disordered Eating Behaviors

**DOI:** 10.7759/cureus.51562

**Published:** 2024-01-03

**Authors:** Joud Makki, Shahad Aljebeli, Shahad Abdulrahman A Alobikan, Bader Altulaihi

**Affiliations:** 1 College of Medicine, King Saud Bin Abdulaziz University for Health Sciences, Riyadh, SAU; 2 Department of Family Medicine and Primary Care, King Abdullah International Medical Research Center, Riyadh, SAU

**Keywords:** eating behaviors, schoolgirls, body image dissatisfaction, adolescent psychology, eat-26, dass-21

## Abstract

Objectives

This study aimed to assess the relationship between depression, anxiety, stress, and disordered eating behaviors among schoolgirls in Riyadh, Saudi Arabia. The current study could provide insights into potential intervention strategies for addressing both aspects simultaneously, contributing to a more holistic approach in the field.

Method

Via simple sampling technique, the current quantitative, descriptive, and cross-sectional study included 347 female adolescents aged between 13 and 18 years in public schools in Riyadh, Saudi Arabia. A self-administered questionnaire comprised demographic information, items on body image satisfaction and perception, the Eating Attitude Test-26 (EAT-26), and the Depression, Anxiety, and Stress Scale-21 (DASS-21).

Results

Among the participants, 32.6% scored at or above the cutoff point on the EAT-26, indicating a negative eating attitude. Additionally, multiple factors such as age, obesity, body image dissatisfaction, and peer pressure were correlated with disordered eating behaviors, with excessive exercise being the most reported weight control behaviors. The prevalence of depression, anxiety, and stress were 58.5%, 73.2%, and 40.9%, respectively. Notably, anxiety was significantly associated with disordered eating behaviors.

Conclusion

In conclusion, disordered eating behaviors were reported by 32.6% of the participants, and symptoms of anxiety were significantly associated with these behaviors. Media consumption appeared to influence more than half of the participants in their weight loss attempts.

## Introduction

Adolescence is a phase during which individuals undergo multiple physio-psycho-social changes. As a result, these changes affect an individual’s self-esteem and perception of body image [1]. Moreover, various factors (such as sociocultural influences, media exposure, and peer pressure) contribute to the self-confidence and eating attitudes of adolescents [2]. Consequently, the development of depressive symptoms, particularly among teenage females, in relation to body shape and outward appearance becomes a potential outcome. Furthermore, unhealthy weight-loss strategies and eating disorders (EDs, such as anorexia nervosa, bulimia nervosa, and binge eating) are associated with severe health-threatening conditions that affect the individual’s physical, reproductive, and emotional well-being [3].

Body image dissatisfaction is characterized by a negative perception of one’s physical appearance that is frequently observed among adolescents, particularly female adolescents. In a large-scale study conducted in the United States, which involved 13,000 adolescents from various ethnic backgrounds to explore the relationship between self-esteem and body image perception, it was found that 25.1% of female participants perceived themselves as overweight, even though their body weight fell within the normal range according to the body mass index (BMI) percentile category [4]. 

In the Middle East, owing to sociocultural influences (including globalization, the widespread media presence, parental influence, and peer pressure), body image dissatisfaction has significantly increased [1,5-7].

EDs are a group of conditions characterized by disturbed eating patterns due to concerns about body weight and shape [8]. EDs have a significant impact on one’s physical, psychosocial, and cognitive functioning due to deficient and problematic dietary intake [9]. In a study that aimed to assess the frequency of disordered eating patterns and methods of weight-controlling behavior among females, it was concluded that 16.5% of the participants had abnormal eating attitudes and 8.5% consumed dieting pills as laxatives in an attempt to achieve the desired body shape [10]. Besides, growing evidence from the United States and Europe suggests increasing the prevalence of disturbed eating behaviors [11,12].

Adolescents, especially female adolescents, are not only vulnerable to the development of disturbed eating attitudes and body image concerns, but they are also at risk of developing psychological problems. Depression is considered a serious public health burden among adolescents [13]. Depression was defined by the World Health Organization as a disorder characterized by loss of pleasure or interest, sadness, feelings of guilt, tiredness, and lack of concentration, which impacts one’s daily activity and academic performance and, in worst cases, might lead to suicide [14]. The effects of depression, anxiety, and stress on students have a huge impact on their academic performance, leading to possible frequent absenteeism and school dropout [15,16].

The alarming psychological consequences linked to disordered eating behaviors and body image dissatisfaction among schoolgirls were considered. Besides, there is a clear lack of locally published studies on the correlation between psychological distress and disordered eating attitudes. The goal of the current study is to assess the relationship between depression, anxiety, stress, and disordered eating behaviors among schoolgirls.

## Materials and methods

Study design, setting, and ethical consideration 

This descriptive cross-sectional study, which was carried out from January 2023 to May 2023, involved female students aged 13-18 years who attended public schools in Riyadh, Saudi Arabia. The study aimed to assess the relationship between depression, anxiety, stress, and disordered eating behaviors among schoolgirls. Therefore, a cross-sectional study design was employed. Prior to data collection, all participants, besides parents and the local educational office, were informed about the scope of the study and its scientific purpose. Ethical approval (NRC22R/365/08 dated 08/23/2022) was obtained from the research committee of King Abdullah International Medical Research Centre (KAIMRC) in Riyadh, Saudi Arabia.

Participants and sampling technique

For the sample size calculation, according to the Saudi General Authority for Statistics, the estimated total population of females within the specified age range was 4 million. Thus, employing a probability of simple sampling technique, the study’s optimal sample size was calculated to be 385, which was adjusted to 400, with a confidence interval of 95% and a margin of error of 5%. For the inclusion criteria, females aged 13-18 years attending secondary or high schools were included. Female students with intellectual disabilities and those who refused to complete the questionnaire were excluded from the study.

Data collection and management

Data collection was carried out by the co-investigators to ensure direct contact with the participants. The self-administered questionnaire used in the study was translated from English to Arabic and was reviewed by experts to ensure accuracy. The questionnaire comprised demographic data, a few questions on body image dissatisfaction and perception, peer pressure, and associated factors to body image dissatisfaction. Additionally, the Arabic versions of the Eating Attitude Test-26 (EAT‑26) tool and Depression, Anxiety, and Stress Scale-21 (DASS-21) were applied in the study, which were previously used and validated in Saudi studies [17,18]. Furthermore, a pilot study was performed on 10% of the sample size, involving 38 participants who were eliminated from the original study sample, to eliminate any potential ambiguities and ensure validity.

The demographic data section of the questionnaire included age, weight, and height. Furthermore, the questionnaire included three statements about self-perception of body image, and students were asked about their satisfaction and self-confidence with their body weight, shape, and size, besides the overall self-esteem of their outer appearance. The perceived peer pressure section included four statements on participants’ self-comparison to peers, the role of peer pressure to lose weight, and the role of media and fashion modeling in an attempt to lose weight. 

The EAT-26 consists of 26 items designed to assess the risk of developing EDs. The tool is widely known and utilized to determine whether the participant has abnormal eating behaviors. The tool is not designed for clinical diagnosis; it only warrants professional intervention due to disturbed eating behaviors. Responses are rated using a 6-point Likert scale. However, three responses are scored as 0 because they represent the non-anorexic ends of the scale, while the remaining three responses are assigned scores of 3, 2, and 1. Thus, the final score ranges from 0 to 78, with a score of 20 and above indicating a high risk of problematic eating behaviors.

The DASS-21 consists of 21 items intended to evaluate the three subscales: (a) depression, (b) anxiety, and (c) stress. Each subscale comprises seven questions and employs a 4-point Likert scale. The scale ranges from 0 to 3, and the final score is multiplied by 2 and then classified based on the DASS manual. The tool is answered based on what the participant felt or happened to her in the last week. The advantages of DASS-21 include self-reporting and straightforward questions suitable for young participants. For the scoring system, the cutoff point for depression is as follows: without symptoms (below 5 points), mild (5-6 points), moderate (7-10 points), severe (11-13 points), and extreme (14 points and above). For anxiety, the cutoff point is as follows: without symptoms (below 4 points), mild (4 points), moderate (5-7 points), severe (8-9 points), and extreme (10 points and above). For stress, the cutoff point is as follows: without symptoms (below 8 points), mild (8-9 points), moderate (10-12 points), severe (13-16 points), and extreme (above 17 points). 

Data analysis

Data was analyzed using the IBM SPSS Statistics, version 28.0 (IBM Corp., Armonk, NY). Frequency and percentage were applied to describe categorical variables, whereas the arithmetic mean, range, and standard deviation (SD) were applied to describe numerical variables. Pearson’s chi-square test was utilized to investigate the association between two categorical variables, with a p-value of less than 0.05 considered as indicative of statistical significance throughout the study.

## Results

**Figure 1:**
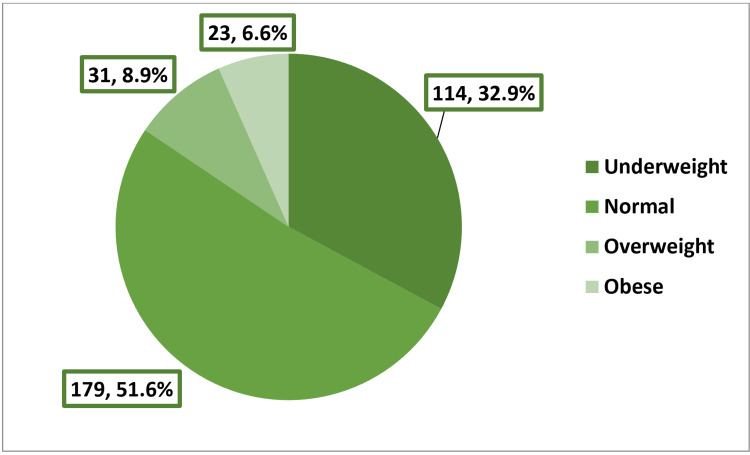
Distribution of body mass index among the participants

The study involved 347 girls, whose ages ranged from 13 to 18 years, with an arithmetic mean of 14.16 and a standard deviation of 1.25 years (Table 1). Figure 1 shows that 32.9% of the girls were categorized as underweight, while 8.9% and 6.6% were classified as overweight or obese, respectively. Most of the participants indicated that they were either sometimes (42.1%) or always (42.3%) satisfied with their body weight. Additionally, most of them were either sometimes (41.8%) or always (49%) satisfied with their body size. Almost two-thirds (61.7%) of the participants reported being always self-confident in their outward appearance (Table 2). A considerable number of girls (44.6%) believed that they were compared to slimmer individuals, while approximately half of them (50.5%) felt that they were subjected to pressure to become slimmer. Over half of the girls believed that the media exerted an influence on their weight loss endeavors (57.1%) and that fashion designs and modeling played a role in shaping their efforts to lose weight (53.3%) (Table 3).

**Table 1:** Age distribution of the participants (n = 347) The data has been represented as mean ± SD. SD, standard deviation

Age in years	Frequency	Percentage
13	136	39.2
14	92	26.5
15	77	22.2
16	23	6.6
17	9	2.6
18	10	2.9
Mean ± SD	14.16 ± 1.25	

**Table 2:** Body image satisfaction among the participants The data has been represented as N (number) and % (percentage).

	Never, N (%)	Sometimes, N (%)	Always, N (%)
Satisfaction with body weight	54 (15.6)	146 (42.1)	147 (42.3)
Satisfaction with body shape and size	32 (9.2)	145 (41.8)	170 (49.0)
Self-confidence with outer appearance	33 (9.5)	100 (28.8)	214 (61.7)

**Table 3:** Participants’ perception of peer pressure regarding their body weight The data has been represented as N (number) and % (percentage).

	Never, N (%)	Rarely, N (%)	Sometimes, N (%)	Often, N (%)	Usually, N (%)	Always, N (%)
People compare between you and other slimmer persons	192 (55.4)	39 (11.2)	26 (7.5)	24 (6.9)	30 (8.6)	36 (10.4)
People exert pressure on you to become slimmer	172 (49.5)	38 (11.0)	32 (9.2)	29 (8.4)	32 (9.2)	44 (12.7)
The media has an influence on your attempts to lose weight	149 (42.9)	29 (8.4)	40 (11.5)	32 (9.2)	43 (12.4)	54 (15.6)
Fashion designs and modeling have influenced your attempt to lose weight	162 (46.7)	38 (11.0)	35 (10.1)	112 (32.3)	0 (0.0)	0 (0.0)

**Figure 2:**
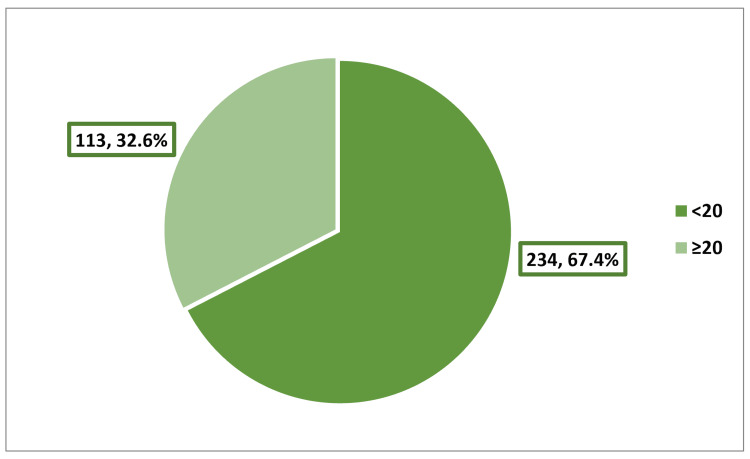
Distribution of the participants according to EAT-26 score EAT-26, Eating Attitude Test-26

The mean EAT-26 score for participants was 15.77 ± 11.55. A total of 113 schoolgirls (32.6%) scored at or above the cutoff point of EAT-26 (20), indicating negative eating attitudes (Figure 2). The highest rate of EDs was observed among 17-year-old girls (66.7%), while the lowest rate was recorded among 13-year-old girls (22.8%), p = 0.022. Girls categorized as obese were more prone to EDs compared to their underweight counterparts (56.5% vs. 26.3%), p = 0.003. Girls expressing perpetual dissatisfaction with their weight, body shape, and outward appearance were more likely to experience EDs compared to girls who were always satisfied with these aspects, p < 0.001. Girls who were frequently or always compared to slimmer individuals, pressured to lose weight, influenced by media to lose weight, and influenced by fashion designs and modeling to lose weight were more prone to EDs compared to those who were never exposed to these pressures, p < 0.001 (Table 4). The most frequently reported body weight-related behavior among the girls in the last six months was exercising for more than 60 minutes a day to either lose or manage weight (44.6%). Following closely was the behavior of binge eating, where the girls felt that they may not be able to stop (35.7%). A smaller proportion of girls reported engaging in vomiting to control weight or shape (9.9%), and an even smaller percentage used laxatives, diet pills, or diuretics for weight or shape control (2.9%). Notably, nearly one-fifth of the participants (19%) reported a weight loss of 20 pounds or more in the past six months (Table 5).

**Table 4:** Factors associated with eating disorders among schoolgirls The data has been represented as N (number) and % (percentage). *p-value is considered significant if p < 0.05, p < 0.001.

		Eating disorders		p-value*
Age in years		No, N = 234 N (%)	Yes, N = 113 N (%)	
	13 (n = 136)	105 (77.2)	31 (22.8)	
	14 (n = 92)	58 (63.0)	34 (37.0)	
	15 (n = 77)	48 (62.3)	29 (37.7)	
	16 (n = 23)	14 (60.9)	9 (39.1)	
	17 (n = 9)	3 (33.3)	6 (66.7)	
	18 (n = 10)	6 (60.0)	4 (40.0)	0.022
Body mass index	Underweight (n = 114)	84 (73.7)	30 (26.3)	
	Normal (n = 179)	125 (69.8)	54 (30.2)	
	Overweight (n = 31)	15 (48.4)	16 (51.6)	
	Obese (n = 23)	10 (43.5)	13 (56.5)	0.003
Satisfaction with body weight	Never (n = 54)	18 (33.3)	36 (66.7)	
	Sometimes (n = 146)	97 (66.4)	49 (33.6)	
	Always (n = 147)	119 (81.0)	28 (19.0)	<0.001
Satisfaction with body shape and size	Never (n = 32)	12 (37.5)	20 (62.5)	
	Sometimes (n = 145)	85 (58.6)	60 (41.4)	
	Always (n = 170)	137 (80.6)	33 (19.4)	<0.001
Self-confidence with outer appearance	Never (n = 33)	12 (36.4)	21 (63.6)	
	Sometimes (n = 100)	58 (58.0)	42 (42.0)	
	Always (n = 214)	164 (76.6)	50 (23.4)	<0.001
People compare between you and other slimmer persons	Never (n = 192)	154 (80.2)	38 (19.8)	
	Rarely (n = 39)	29 (74.4)	10 (25.6)	
	Sometimes (n = 26)	14 (53.8)	12 (46.2)	
	Often (n = 24)	7 (29.2)	17 (70.8)	
	Usually (n = 30)	15 (50.0)	15 (50.0)	
	Always (n = 36)	15 (41.7)	21 (58.3)	<0.001
People exert pressure on you to become slimmer	Never (n = 172)	130 (75.6)	42 (24.4)	
	Rarely (n = 38)	27 (71.1)	11 (28.9)	
	Sometimes (n = 32)	22 (68.8)	10 (31.3)	
	Often (n = 29)	17 (58.6)	12 (41.4)	
	Usually (n = 32)	21 (65.6)	11 (34.4)	
	Always (n = 44)	17 (38.6)	27 (61.4)	<0.001
The media has an influence on your attempts to lose weight	Never (n = 149)	119 (79.9)	30 (20.1)	
	Rarely (n = 29)	21 (72.4)	8 (27.6)	
	Sometimes (n = 40)	24 (60.0)	16 (40.0)	
	Often (n = 32)	22 (68.8)	10 (31.3)	
	Usually (n = 43)	27 (62.8)	16 (37.2)	
	Always (n = 54)	21 (38.9)	33 (61.1)	<0.001
Fashion designs and modeling have influenced your attempt to lose weight	Never (n = 162)	137 (84.6)	25 (15.4)	
	Rarely (n = 38)	26 (68.4)	12 (31.6)	
	Sometimes (n = 35)	24 (68.6)	11 (31.4)	
	Often (n = 112)	47 (42.0)	65 (58.0)	<0.001

**Table 5:** Body weight-related behavior in the last 6 months among the participants The data has been represented as N (number) and % (percentage).

	Never, N (%)	≤Once/month, N (%)	2-3 times/month, N (%)	Once/week, N (%)	2-6 times/week, N (%)	Daily, N (%)
Gone on eating binges where I feel that I may not be able to stop	223 (64.3)	58 (16.7)	28 (8.1)	15 (4.3)	16 (4.6)	7 (2.0)
Ever vomited to control weight or shape	313 (90.1)	15 (4.3)	4 (1.2)	4 (1.2)	2 (0.6)	9 (2.6)
Ever used laxatives, diet pills, or diuretics to control weight or shape	337 (97.1)	4 (1.2)	1 (0.3)	0 (0.0)	0 (0.0)	5 (1.4)
Exercised more than 60 minutes a day to lose or control weight	192 (55.4)	47 (13.5)	23 (6.6)	27 (7.8)	24 (6.9)	34 (9.8)
	No			Yes		
Lost 20 pounds or more in the past 6 months	281 (81.0)			66 (19.0)		

**Figure 3:**
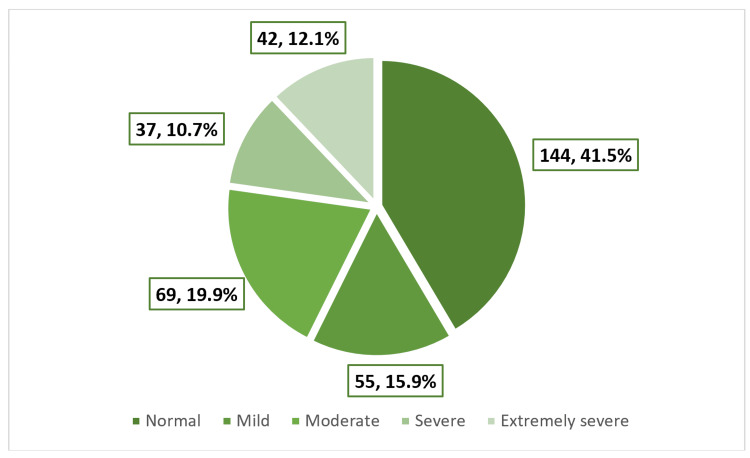
Prevalence and severity of depression among the participants

**Figure 4:**
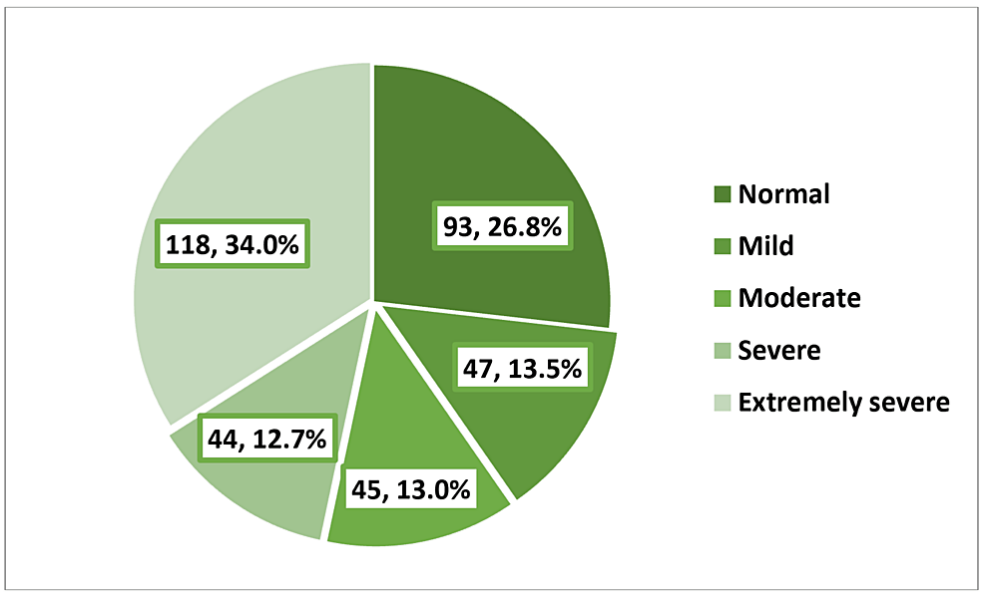
Prevalence and severity of anxiety among the participants

**Figure 5:**
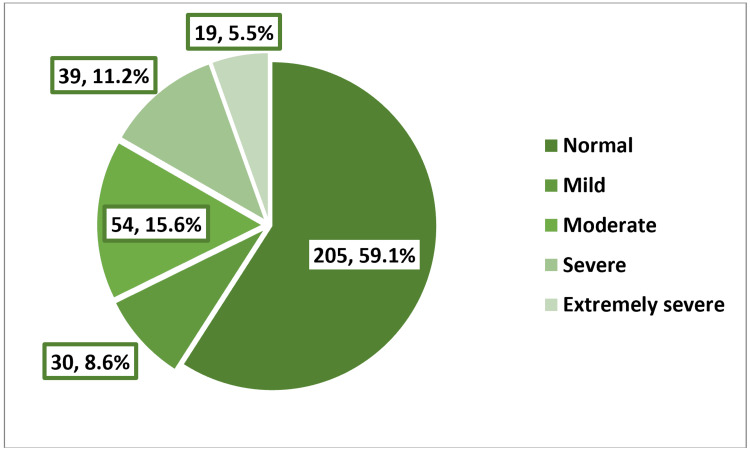
Prevalence and severity of stress among the participants

As shown in Figure 3, over half of the girls (58.5%) experienced feelings of depression, with 10.7% and 12.1% falling into the categories of severe or extremely severe depression, respectively. Among girls with EDs, 15.9% and 21.2% exhibited severe or extremely severe depression, respectively, in contrast to only 8.1% and 7.7% among those without EDs, p < 0.001 (Table 6). As shown in Figure 4, the prevalence of anxiety was 73.2%, with 12.7% of the girls experiencing severe anxiety and 34% reporting extremely severe anxiety. Among girls with EDs, 54% had extremely severe anxiety, while this proportion was 24.4% among those without EDs, p < 0.001 (Table 7). As shown in Figure 5, the prevalence of stress was 40.9%, with 11.2% of the girls experiencing severe stress and 5.5% reporting extremely severe stress. Girls with EDs exhibited higher levels of severe (20.4%) and extremely severe (10.6%) stress compared to those without EDs, who reported rates of 6.8% and 3%, respectively, p < 0.001 (Table 8).​​​​​​​​​​​​​​

**Table 6:** Association between depression and eating disorders among schoolgirls The data has been represented as N (number) and % (percentage). Pearson chi-square = 39.18; degree of freedom = 4; p < 0.001

Depression	Eating disorders	
	No, N = 234 N (%)	Yes, N = 113 N (%)
Normal (n = 144)	120 (51.3)	24 (21.3)
Mild (n = 55)	40 (17.1)	15 (13.3)
Moderate (n = 69)	37 (15.8)	32 (28.3)
Severe (n = 37)	19 (8.1)	18 (15.9)
Extremely severe (n = 42)	18 (7.7)	24 (21.2)

**Table 7:** Association between anxiety and eating disorders among schoolgirls The data has been represented as N (number) and % (percentage). Pearson chi-square = 33.56; degree of freedom = 4; p < 0.001

Anxiety	Eating disorders	
	No, N = 234 N (%)	Yes, N = 113 N (%)
Normal (n = 93)	76 (32.5)	17 (15.0)
Mild (n = 47)	38 (16.2)	9 (8.0)
Moderate (n = 45)	34 (14.5)	11 (9.7)
Severe (n = 44)	29 (12.4)	15 (13.3)
Extremely severe (n = 118)	57 (24.4)	61 (54.0)

**Table 8:** Association between stress and eating disorders among schoolgirls The data has been represented as N (number) and % (percentage). Pearson chi-square = 43.93; degree of freedom = 4; p < 0.001

Stress	Eating disorders	
	No, N = 234 N (%)	Yes, N = 113 N (%)
Normal (n = 205)	165 (70.5)	40 (35.4)
Mild (n = 30)	14 (6.0)	16 (14.2)
Moderate (n = 54)	32 (13.7)	22 (19.5)
Severe (n = 39)	16 (6.8)	23 (20.4)
Extremely severe (n = 19)	7 (3.0)	12 (10.6)

## Discussion

Body image dissatisfaction, characterized by a negative perception of one’s physical appearance, and disturbed eating behavior are interconnected phenomena frequently observed among adolescents, particularly among female adolescents. These phenomena are attributed to the biophysical changes that occur during puberty, during which the female body tends to accumulate fat [19-21]. Disturbed eating behavior includes multiple abnormal eating attitudes such as self-induced vomiting, strict diet control, binge eating, and the use of laxatives or diuretics [22]. Several risk factors have been shown to be associated with disturbed eating behavior, including peer pressure, social media usage, and sociocultural influences [23]. Early recognition of disturbances in eating attitude is crucial for preventing the development of severe EDs and psychiatric conditions, which were identified in previous studies to be associated with such phenomena [23-25]. 

The findings of this study, which involved 347 adolescent schoolgirls with a mean age of 14.16 and a standard deviation of 1.25 years, revealed that over half of the participants had a normal BMI, while fewer than 16% were classified as overweight or obese. Furthermore, nearly two-thirds of the participants expressed self-confidence in their outward appearance. This finding is supported by a similar study conducted by Almuhlafi et al., involving 399 subjects, which indicated that 74% of the participants had a normal BMI, with 16.3% being overweight or obese [26]. Additionally, 68% of the participants in that study were confident in their outward appearance [26]. This result can be explained by the fact that female adolescents during this phase of development often engage in risky eating behaviors to lose weight and bolster self-confidence regarding their appearance, particularly under the influence of media pressure and beauty standards. 

Peer pressure greatly affects one’s perceived body image and outward appearance. Peers may not always provide the necessary support or concern for the developing adolescent, leading to the emergence of various forms of disturbed eating behaviors. The findings of this study indicate that half of the girls felt pressure from those around them to achieve a slimmer physique. This observation agrees with a study by Mousa et al., which established a connection between negative comments on physical appearance and body image dissatisfaction [27]. It is not surprising that media, fashion trends, and modeling greatly influence girls and their attempts to lose weight and achieve the prevailing standards of feminine beauty. This study reveals that more than half of the participants acknowledged being influenced by media, fashion trends, and modeling in their pursuit to lose weight. This phenomenon is substantiated by numerous studies, including one recently published in 2023. This particular study aimed to explore the relationship between social media usage, body image dissatisfaction, and disturbed eating behavior, with a specific focus on the increased consumption of social media content during the COVID-19 pandemic. The study’s findings underscored a substantial prevalence of body image dissatisfaction and disordered eating behavior among users of image-based platforms such as Snapchat, TikTok, and YouTube [28]. 

In this study, EAT-26, a screening tool for identifying disordered eating patterns, was employed to estimate the prevalence of such behavior. The study revealed a prevalence rate of EDs at 32.6%. Numerous factors were associated with negative eating attitudes, including age, obesity, body image dissatisfaction, and perceived peer pressure. Specifically, the highest incidence of EDs was noted among girls aged 17 years. This finding is supported by a similar study that was conducted in the northern region of Saudi Arabia, where the participants aged 17-18 years and scoring above the EAT-26 cutoff point exhibited similar patterns [29]. This intriguing observation can be attributed to the fact that postmenarchal girls are more prone to developing body image dissatisfaction and disturbed eating attitudes compared to premenarchal females [30,31]. Obesity is another factor related to disordered eating patterns. Multiple studies have identified this correlation, including research by Jones and Rodin, which demonstrated that overweight and obese girls tended to have an increased risk of developing disturbed eating attitudes compared to underweight participants [32]. Furthermore, body image dissatisfaction is another influential factor in disturbed eating patterns. A recent study conducted in the southwestern region of Saudi Arabia aimed to estimate the prevalence of body image dissatisfaction. The above-mentioned study revealed that a considerable portion of participants expressed a desire to become thinner, with only a minority seeking weight gain [33]. Consequently, the absence of education regarding proper dietary practices and an individual’s negative self-perception contribute to the emergence of disordered eating attitudes. Moreover, perceived peer pressure also demonstrated a significant correlation with disturbed eating attitudes. This correlation is logical because peers’ expectations and perceptions can act as potential stressors for females who are navigating the process of forming their identities during this crucial stage of development.

While frequent exercise is often recommended to alleviate dysmenorrhea symptoms, numerous studies have established a link between excessive exercise and menstrual irregularities, including lack of ovulation and delayed menarche [34]. The findings of this study showed that the most prevalent weight-controlling behavior among participants was exercising for more than 60 minutes in an attempt to lose weight, followed by binge eating, deliberate vomiting, and the use of dieting pills. This contrasts with the study by Jones and Rodin, which identified diet control as the most frequent weight-controlling behavior, followed by binge eating [32].

Adolescents, particularly female adolescents, during these life stages, are not only susceptible to developing EDs, but they are also at risk of developing mental health issues [13]. In this study, the DASS-21 tool was used to assess the levels of depression, anxiety, and stress among the female participants. The study revealed that the prevalence of depression, anxiety, and stress was 58.5%, 73.2%, and 40.9%, respectively. A recently published study that aimed at assessing the prevalence and correlation between depression, anxiety, stress, and disturbed eating patterns among college students reported a prevalence of 29.3%, 55%, and 21.6%, respectively [35]. Furthermore, the current study found a significant association between disturbed eating attitudes and anxiety, followed by depression, with stress being the least reported. This observation agrees with previous findings by Nadeem et al. [36].

The study suggests a significant association between disordered eating behaviors and heightened symptoms of depression, anxiety, and stress in schoolgirls. Therefore, educating teenage girls by a healthcare provider or parents is crucial to prevent psychological problems and reduce the associated complications from an early stage. The methods of educating female adolescents can be diverse and encompass various approaches. Among these, utilizing image-based platforms, such as Snapchat, TikTok, and YouTube, holds promise because teenagers are the primary audience for such platforms. 

The current study contributes to the existing literature by shedding light on the nuanced relationship between disordered eating and mental health in a specific population, i.e., schoolgirls. The findings align with previous studies highlighting the interconnectedness of mental health and eating behaviors. Additionally, the study could provide insights into potential intervention strategies for addressing both aspects simultaneously, contributing to a more holistic approach in the field. Limitations of the study include the small sample size and self-reported questionnaire. Thereupon, future studies with larger sample sizes and nationwide representation are warranted.

## Conclusions

Our findings indicate that a considerable proportion of female school adolescents were either sometimes or always satisfied with their body weight despite having high rates of negative peer pressure regarding body weight. Nearly half of the participants reported being compared to slimmer individuals, while a similar proportion felt pressure to attain a slimmer physique. Sociocultural factors can significantly affect self-esteem and contribute to heightened body dissatisfaction. In addition, this study showed that media pressure plays a considerable role in body perception, with over half of the girls reporting that media influenced their attempts to lose weight. Thus, decreasing social media usage may positively affect self-esteem. The highest rate of EDs was observed among obese girls. Therefore, it is speculated that lifestyle modification may enhance body satisfaction. In terms of psychological well-being, participants with EDs exhibited considerably higher proportions of extremely severe depression, anxiety, and stress. Taking into account this information, it is important to emphasize the significance of body dissatisfaction, EDs, and mental health in future studies.
